# Karyotypic description of the stingless bee *Oxytrigona* cf. *flaveola* (Hymenoptera, Apidae, Meliponina) of a colony from Tangará da Serra, Mato Grosso State, Brazil

**DOI:** 10.1590/S1415-47572010000300020

**Published:** 2010-09-01

**Authors:** Diones Krinski, Anderson Fernandes, Marla Piumbini Rocha, Silvia das Graças Pompolo

**Affiliations:** 1 Departamento de Ciências Biológicas, Universidade do Estado de Mato Grosso, Tangará da Serra, MTBrazil; 2 Departamento de Morfologia, Universidade Federal de Pelotas, Pelotas, RSBrazil; 3 Departamento de Biologia Geral, Universidade Federal de Viçosa, Viçosa, MGBrazil

**Keywords:** chromosome, heteromorphism, fluorochrome

## Abstract

The aim was to broaden knowledge on the cytogenetics of the subtribe Meliponina, by furnishing cytogenetic data as a contribution to the characterization of bees from the genus *Oxytrigona*. Individuals of the species *Oxytrigona* cf. *flaveola*, members of a colony from Tangará da Serra, Mato Grosso State, Brazil, were studied. The chromosome number was 2n = 34, distributed among four chromosomal morphologies, with the karyotype formula 8m+8sm+16st+2t. Size heteromorphism in the first metacentric pair, subsequently confirmed by sequential staining with fluorochrome (DA/DAPI/CMA_3_ ), was apparent in all the examined individuals The nucleolar organizing regions (NORs) are possibly located in this metacentric chromosome pair. These data will contribute towards a better understanding of the genus *Oxytrigona*. Given that species in this group are threatened, the importance of their preservation and conservation can be shown in a sensible, concise fashion through studies such as this.

The number of stingless bee species (subtribe Meliponina) found in the Neotropics is extremely high, with approximately 400 known to date (Biesmeijer and Slaa, 2004). In Brazil, there are 192 already described species, belonging to 27 genera (Silveira *et al.*, 2002). Studies have reached the cytogenetic level in 75, whereas in many only the chromosome number has been determined (Rocha *et al.*, 2003). Thus, the urgent need for further studies, as many native species of social bees are becoming extinct, through the destruction of their habitats by deforestation, forest fires, the lumber industry, insecticides and honey collectors (Kerr *et al.*, 2001).

Certain groups of meliponines, such as the genus *Oxytrigona*, have specific characteristics. Bees of this genus are commonly known as “*cospe-fogo*” (*fire spitting*), due to the peculiar characteristic of secreting a caustic liquid (formic acid) from the mandibular glands, thereby giving rise to severe burns in both animals and humans, while leaving permanent spots on the skin. Besides being highly aggressive, they are also cleptobiotic, as colony robbers of other meliponine species (Roubik *et al.*, 1987; Roubik, 1992; Souza *et al.*, 2007).

The genus was last reviewed by Schwarz (1948), who only recognized one species, *Oxytrigona tataira*, yet it is now considered to include several subspecies and even undescribed species (Silveira *et al.*, 2002). Michener (2000) reported the existence of eight species of *Oxytrigona* in the Neotropics, six of which were found in Colombia (Nates-Parra, 2001), during a study on local stingless bees. Recently, Gonzalez and Roubik (2008) reviewed the genus and described 11 species of *Oxytrigona*.

Among those species of *Oxytrigona* so far studied cytogenetically, only the chromosome umber of *O. tataira* (n = 17) was placed in evidence by the crushing technique, thereby revealing four morphological types of chromosomes, classified in decreasing order based on size (Kerr, 1972).

In the state of Mato Grosso (Brazil), the cytogenetic study of bees as a whole, is rare (Costa *et al.*, 2004). Therefore, there is a need for a cytogenetic study on *Oxytrigona* cf. *flaveola*, which is found in this region, especially considering the current threat of extinction to approximately 100 bee species, as emphasized by Kerr *et al.* (1996).

Further studies of these bees would contribute towards the characterization and correct classification of species. Cytogenetic analysis is a resource that, together with other areas of research, has offered contributions to knowledge on phylogeny (Costa *et al.*, 2003; Camargo and Pedro, 2003; Rocha *et al.*, 2003; Rasmussen and Cameron, 2007; Gonzalez and Roubik 2008), speciation mechanisms (Tavares *et al.*, 2007; Lopes *et al.*, 2008; Souza *et al.*, 2008) and genetic variability (Rocha *et al.*, 2002; Domingues *et al.*, 2005; Martins *et al.*, 2009), seeing that chromosomes are the physical basis of the genetic system.

A colony of *O.* cf. *flaveola* was collected from a wall in the urban area of Tangará da Serra (14°37'42” S, 57°29'53” W), Mato Grosso State, Brazil, to be used for cytogenetic analysis. Voucher specimens are deposited in the Biology Laboratory of the Universidade do Estado de Mato Grosso, Tangará da Serra *campus*. The material used to obtain metaphase chromosomes was extracted from the cerebral ganglia of 20 post-defecating *O.* cf. *flaveola* larvae*,* according to the methodology developed by Imai *et al.* (1988). A minimum of 10 metaphases per specimen were analyzed.

Conventional staining was carried out with a solution of 1 mL of Giemsa, in 30 mL of Sörensen buffer 0.06 M (pH = 6.8) for 25 min at room temperature, followed by sequential staining with fluorochromes (4'-6-diamidino-2-phenylindole - DAPI and chromomycin A_3_ - CMA_3_) (Schweizer, 1980). 4'-6-diamidino-2-phenylindole (DAPI) is a fluorochrome that binds to AT and GC bases. Nevertheless fluorescence intensity is significantly higher with DNA rich in AT bases, thereby generating more pronounced, brilliant regional banded patterns. Chromomycin A_3_ (CMA_3_) is an antibiotic with affinity for GC base pairs (Sumner, 1990). Furthermore, CMA_3_ regions are generally associated with nucleolar organizer regions (NORs).

Metaphase cells revealed by Giemsa and fluorochrome staining were captured by a CCD camera (OPTRONICS, model DEI-470) connected to an Olympus TM BX60 microscope equipped with epifluorescence, with a WB filter (λ = 450-480 nm) and immersion objectives at 100x magnification. Graphs and karyograms were constructed using an image analysis program (Image-Pro^®^ Plus, version 3.1, Media Cybernetics, 1998).

*Oxytrigona* cf. *flaveola* proved to have 2n = 34 chromosomes (Figure 1a), as previously observed in *O. tataira* (Kerr, 1972). The four morphological chromosome types were determined based on nomenclature as proposed by Levan *et al.* (1964): four metacentric pairs (m), four submetacentric pairs (sm), eight subtelocentric pairs (st) and one telocentric pair (t) for diploid individuals, thereby furnishing the karyotype formula 8m+8sm+16st+2t (Figure 1a). Size heteromorphism was found in the first chromosome pair of all the individuals analyzed (Figure 1a, b). *Oxytrigona* cf. *flaveola* has a higher chromosome number than previously karyotyped species belonging to the tribe Meliponini (Pompolo, 1992; Rocha and Pompolo, 1998; Rocha *et al.*, 2002, 2003), with most chromosomes being either submetacentric or subtelocentric. Studies on 27 genera of the tribe gave note of haploid chromosome numbers ranging from 8 to 20 chromosomes (Kerr, 1948, 1952, 1969, 1972; Kerr and Silveira, 1972; Hoshiba, 1988, Hoshiba and Imai, 1993; Pompolo, 1994; Brito-Ribon *et al.*, 1999).

**Figure 1:**
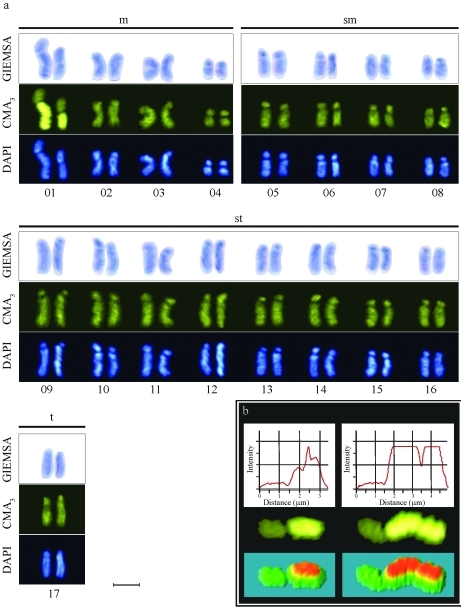
Mitotic chromosomes of *Oxytrigona* cf. *flaveola* sequentially stained with (a) Giemsa, CMA_3_ and DAPI. The karyotype presented four metacentric pairs, four submetacentric, eight subtelocentric and one telocentric for diploid individuals. (b) Heteromorphism of fluorescence intensity and the size of the arm preferentially stained by fluorochrome CMA_3_ in the first chromosome pair. Scale bar: 5 μm.

The high chromosome number of *O.* cf. *flaveola* may be related to centric fission, as proposed by the theory of minimal interaction (Imai *et al.*, 1988). According to this theory, the karyotype evolved as a means of minimizing genetic damage through centric fission, with the consequential increase in chromosome number. The regions of fission would correspond to an unstable telomeric region. Chromosome stability would be regained with *in tandem* growth of regional heterochromatin, thereby generating heterochromatic arms.

Sequential staining showed that chromosomes of pair 1 are preferably CMA_3_^+^. These chromosomes had one arm preferentially stained by CMA_3_ fluorochromes, usable for revealing size heteromorphism (Figure 1b). Brito *et al.* (2003) reported CMA_3_^+^ heteromorphic markings found in the large chromosomes of some species of *Partamona*, a possible indication of phylogenetic relationship between the genera *Partamona* and *Oxytrigona*, as suggested by Costa *et al.* (2003).

There are two hypotheses for explaining heteromorphism in pair one: (1) the small chromosome could be an ancestral condition, with the larger originating through *in tandem* amplification of regions rich in GC pairs (CMA_3_^+^); and (2) the large chromosome would be the ancestral condition, with the smaller originating from deletion of a portion of the former. Thus, cytogenetic analysis of other *O.* cf. *flaveola* colonies might provide relevant data to prove either of the two hypotheses.

GC bases were prevalent in the region marked by CMA_3_ in pair 1 of *O.* cf. *flaveola*, thereby implying that this chromosome may contain sites of ribosomal DNA sequences, since there is generally an association between the presence of nucleolar organizer regions (NORs) with CMA_3_ labeling in the same chromosome region (Sumner, 1990).

A positive correlation between CMA_3_ and NORs has been reported in several species of the subtribe Meliponina, as *Partamona mulata* and *Partamona vicina* (Brito-Ribon *et al.*, 1999), *Partamona peckolti* (Brito *et al.*, 2003), *Partamona helleri* and *Partamona seridoensis* (Brito *et al.*, 2005), *Trigona fulviventris* (Domingues *et al.*, 2005), and four other species of *Trigona* (Costa *et al.*, 2004).

The scarcity of biological information on bees from the subtribe Meliponina, especially the genus *Oxytrigona*, underlines the importance of further cytogenetic knowledge of this group, as a whole. This could be useful in orientating taxonomy and conservation methods. Cytogenetics directly affects progress in taxonomy studies, by ensuring biological data with the elimination of subjectivity in systematic classification, especially in the Meliponina, through numerical taxonomy (Kerr *et al.*, 1967).

The information obtained in this work, besides being of use in future cytotaxonomic studies, will be of assistance in comparative analyses, as a means of clarifying both taxonomic problems and those phenomena involved in karyotype evolution in this group.
